# The mediating role of narcissism in the effects of regulatory mode on positivity

**DOI:** 10.1007/s12144-021-02014-w

**Published:** 2021-06-26

**Authors:** Daniela Di Santo, Calogero Lo Destro, Conrad Baldner, Alessandra Talamo, Cristina Cabras, Antonio Pierro

**Affiliations:** 1 Department of Social and Developmental Psychology, Sapienza University of Rome, Via dei Marsi, 78, 00185 Rome, Italy; 2 Niccolò Cusano University, Rome, Italy; 3 University of Cagliari, Cagliari, Italy

**Keywords:** Regulatory mode, Narcissism, Adaptive, Maladaptive, Positivity

## Abstract

Positivity (i.e., the individual tendency to positively approach life experiences) has proven to be an effective construct applied in positive psychology. However, individuals’ self-regulation may have contrasting effects on positivity. We specifically examined whether positivity could be partially explained through two aspects of motivation concerned with self-regulation: *locomotion* (i.e., a motivational orientation concerned with movement) and *assessment* (i.e., a motivational orientation concerned with comparison and evaluation). Furthermore, based on previous literature that found a link between these aspects and narcissism, we examined whether “adaptive” and “maladaptive” dimensions of narcissism could mediate the effects of locomotion and assessment on increased or decreased positivity. Narcissism was defined by previous research as adaptive or maladaptive insofar as it leads or does not lead to increased psychological well-being. We estimated a mediation model with multiple independent variables and multiple mediators in a cross-sectional study with self-reported data from 190 university students. We found that both locomotion and assessment were associated with adaptive narcissism, which in turn was positively associated with positivity. However, assessment was also associated with maladaptive narcissism, which in turn was negatively associated with positivity. Relationships between aspects of self-regulation, narcissism, and positivity can have significant implications which will be discussed.

## Introduction

The tendency of individuals to view and address life and experience with a positive outlook (i.e., “positivity”; Caprara et al., 2012) is a crucial variable in psychological adjustment with positive benefits for one’s life. Positivity is defined as “as an individual propensity to positively evaluate, or to be positively oriented toward various life domains including oneself, and one’s future and past experiences” (Caprara et al., 2009, p. 277). This positive mindset therefore implies seeing life events through a positive lens and having the skills and personal resources to cope with adversity, loss, and failure throughout life (Caprara et al., 2012). For example, and of current interest, positivity has recently been shown to be a psychological resource that can potentially protect people from the negative mental effects of Covid-19 (Yıldırım & Güler, 2021).

Despite the growing interest in this construct, we still do not know much about the factors—such as characteristics, predispositions, and beliefs—that might promote it. Yet, it is worth learning more, given the important biological and social functions performed by positivity (Caprara et al., 2016; Tisak, 2019). Personality traits and beliefs of self-efficacy have been examined so far by the research (see Caprara et al., 2019, for a review). Given the conflicting research surrounding narcissism (e.g., Morf & Rhodewalt, 2001), we investigated whether narcissism could be associated with both higher and lower positivity, dependent on the individual’s active self-regulation goals.

It is suggested that self-regulation, or the process by which people evaluate, direct and control their means and actions towards goals, is linked to how people cope with difficulties (Aspinwall, 2001), but little is known about the effects of self-regulatory orientations on positive outlook. We refer to two self-regulatory aspects, both required for the successful goal pursuit according to the Regulatory Mode Theory (RMT; Higgins et al., 2003; Kruglanski et al., 2000), namely *locomotion and assessment* regulatory modes: the first mode is concerned for managing movement from state to state, such that the change of state takes place; the second mode is concerned for comparison and critical evaluation of alternative goals and means options, such that the right (or best) goal and the right (or best) means to pursue it are chosen (Higgins et al., 2003). The first research question is whether and how regulatory modes have any effect on positivity. Relying on previous literature and findings on the regulatory modes (e.g., Higgins et al., 2003; Kruglanski et al., 2000) that a high mode of “acting” and “moving” leads to positive feelings as opposed to “chronically comparing”, positivity could be stimulated by locomotion regulatory mode and hampered by assessment regulatory mode.

However, consistent with the idea that self-regulation is central to narcissism (Morf & Rhodewalt, 2001), research has shown that regulatory modes may also underlie different narcissistic feelings (Boldero et al., 2015). In turn, narcissism affects various individuals’ well-being outcomes (Clarke et al., 2015). It is thus possible that narcissism could mediate the effect of the regulatory modes on positivity. In other words, depending on the type of narcissism experienced (see also Boldero et al., 2015) by locomotors (i.e., individuals who have a high locomotion orientation) and assessors (i.e., individuals who have a high assessment orientation), the effects of regulatory modes on positivity may vary. Accordingly, given its multidimensional nature, narcissism is presumed to have opposite effects on well-being outcomes, depending on its “adaptive” or “maladaptive” nature (e.g., Clarke et al., 2015). An aim of the present work was to examine different dimensions of narcissism in mediating the effects of regulatory modes on positivity. For this purpose, we theorize a distinct mediational model involving both regulatory modes (locomotion and assessment), narcissism, and positivity. We tested this integrated model in a non-clinical university student sample.

In the next sections, we will present the relevant literature on the variables of interest and our hypotheses. After this, we will present the method, analyses, and results of the study. Next, a general discussion will be implemented which also involves limitations and implications for future research. The work will be ended with general conclusions.

### Regulatory Mode and Positivity

Assessment and locomotion modes reflect two different aspects of self-regulation, the first being comparative in nature, the second being concerned with the movement from state to state (Kruglanski et al., 2000). Since a focus on self-evaluation can highlight discrepancies between one’s actual self as a state and the desired self as an alternate state (Higgins, 1987), assessment mode, which is involved in perennial self-evaluation, ruminates on this discrepancy (Higgins et al., 2003; Kruglanski et al., 2000). Accordingly, strong assessors tend to generally experience negative affect, lower optimism and self-esteem (Kruglanski et al., 2000), and higher work stress (Lo Destro et al., 2017). On the other hand, locomotion mode reflects the self-regulatory aspect concerned with initiating and maintaining action that will reduce the discrepancy between one’s current state and the desired end state (Higgins et al., 2003; see also Kruglanski et al., 2016); consistently, high locomotors generally experience positive affect, optimism, and self-esteem (Kruglanski et al., 2000), low work stress (Lo Destro et al., 2017; Lo Destro et al., 2018), low hopelessness, and high subjective well-being (Di Santo et al., 2018; Di Santo et al., 2020).

Consistent with this literature, locomotion mode, rather than assessment mode, should have a general positive attitude towards self and life, operationalized as a higher-order factor that captures both satisfaction with living conditions, positive expectations about the future (e.g., optimism), and self-esteem (Caprara et al., 2012). Such general positivity factor is seen by researcher as a determinant of one’s health and well-being (Caprara et al., 2016; Caprara et al., 2019). In examining their relationship to other factors, research has found that certain personality traits (extroversion and neuroticism) predict positivity, which, in turn, mediates their relationship with subjective happiness (Lauriola & Iani, 2015); positive thinking has also been shown to be promoted by affective and social self-efficacy beliefs, or people’s beliefs in their perceived ability to successfully manage affectivity and interpersonal relationships (see Caprara et al., 2019, for a review). We assume that positivity could be stimulated in the opposite way by the two regulatory modes, i.e., positively having a high locomotion and negatively having a high assessment. However, given the associations previously found between regulatory modes and narcissism (Boldero et al., 2015; Hanke et al., 2019), the content of narcissism experienced by locomotors and assessors could also partially explain the level of positivity they feel, insofar as narcissism and well-being are related in a multifaceted way. This will be briefly discussed in the next section.

### Narcissism as a Mediator

Literature in clinical and personality psychology has widely supported a multidimensional conceptualization of narcissism (e.g., Miller et al., 2011; Pincus et al., 2009; Wink, 1991). Accordingly, Clarke et al. (2015) identified eight dimensions that reflected adaptive or maladaptive content of narcissism, corresponding to: (1) Leadership/Authority, (2) Superiority, (3) Grandiose Exhibitionism, (4) Contingent Self-Esteem, (5) Devaluing the Self, (6) Grandiose Fantasy, (7) Manipulative, and (8) Entitlement. Of such dimensions, Clarke et al. (2015) suggests that Leadership/Authority and Superiority would reflect adaptive narcissism, as they were positively correlated with self-esteem, and negatively correlated with neuroticism. Furthermore, although in some cases exhibitionism was suggested to be associated with poor adjustment (Raskin & Terry, 1988), in the study by Clarke et al. (2015) Grandiose Exhibitionism was positively associated with self-esteem and not significantly associated with psychological distress. Contingent Self-Esteem and Devaluing the Self would reflect maladaptive narcissism, as they positively predicted depression and stress, and negatively predicted self-esteem (Clarke et al., 2015).

We mainly relied on these opposite effects on psychological well-being outcomes (Clarke et al., 2015; see also Cai & Luo, 2018) to help define the research hypotheses. Although we explore each of the dimensions of narcissism identified by Clarke et al. (2015), we therefore focus our hypotheses on those more of adaptive and maladaptive narcissism in predicting that they are positively and negatively associated with positivity, respectively. The distinction of maladaptive and adaptive narcissism is in accordance with past literature (Raskin & Terry, 1988) suggesting that only certain aspects of narcissism are associated with maladjustment. Accordingly, facets of adaptive narcissism (i.e., narcissistic superiority) were found to predict life satisfaction (Miller et al., 2019). A link was observed, instead, between more maladaptive narcissistic traits and depressive traits (Tritt et al., 2010). Individuals may also present a specific vulnerability to pathological and maladaptive forms, as research (Engel-Yeger et al., 2016) that has found stable extreme sensory processing patterns as unique sensory profiles in individuals with depression and mood disorders.

Moreover, consistent with previous findings that see locomotion and assessment as self-regulatory underpinnings of grandiose and vulnerable narcissism (Boldero et al., 2015), we hypothesize that both modes can develop narcissistic factors. Specifically, our hypothesis on locomotion mode is that the concern with “making something happen” (Higgins et al., 2003) could develop factors of adaptive narcissism that facilitate the movement goals of people characterized by a locomotion mode; this would, in turn, increase positivity. This is consistent with a view supported by empirical findings (Kruglanski et al., 2016), that locomotion mode generally promotes positive health outcomes. We thus expect that the positive relation between locomotion and positivity will be mediated by adaptive dimensions of narcissism (H1).

The hypothesis on assessment mode is more complex. People characterized by an assessment mode need to consider different options in order to make the best choice (Higgins et al., 2003). This mode has clear benefits, as people can perceive more assurance in their choices; however, they can also subject themselves and their choices to increased criticism. These two possibilities reflect two sides of narcissism: increased self-assurance reflects aspects of adaptive narcissism whereas increased self-criticism reflects maladaptive narcissism. As argued by Boldero et al. (2015), the successes and failures encountered in trying to be “right” can change the nature of assessors’ feelings. Accordingly, assessment could have a double relationship with narcissism, i.e., assessors could be both adaptive and maladaptive narcissists, and this ambivalence can be reflected in the positivity they experience as well.

We could therefore expect that the concern with “making the right or best choice”, which reflects greater assurance in one’s choices, may develop adaptive narcissism factors; this would, in turn, increase positivity. Our hypothesis is that the positive relation between assessment and positivity will be mediated by adaptive dimensions of narcissism (H2). The assumption that both locomotion and assessment modes can be associated with adaptive narcissism is consistent with previous findings on the positive effects of the regulatory mode conjunction (i.e., the presence of both modes; Pierro et al., 2018). However, we might at the same time expect that concern with doing the right thing reflecting an ongoing evaluation of the discrepancies between their current and desired state (Kruglanski et al., 2000) can develop maladaptive narcissistic factors and, in turn, decrease positivity. Our third hypothesis is that the negative relationship between assessment and positivity will be mediated by maladaptive dimensions of narcissism (H3).

In sum, we expect that locomotion mode should have a positive relationship with adaptive narcissism and, therefore, also with positivity. The assessment effect should be ambivalent, in that it can predict both increased and decreased positivity, dependent on the dimension of narcissism—adaptive or maladaptive—that mediates the relationship. These assumptions are tested in the study described below.

## Method

### Participants & Procedure

One hundred ninety students in the master’s degree program in Psychology (150 females, 40 males; *M*_*age*_ = 24.93, *SD*_*age*_ = 4.396) at a large public Italian university participated in this research on a voluntary basis. A total of 190 students were invited in the study and replied our survey (response rate of 100%). The study complied with the Declaration of Helsinki and was approved by the departmental Ethical Committee under protocol 1249, titled “Analysis of the relationship between Regulatory Modes and Positivity.” Participants completed a paper-and-pencil questionnaire comprising the set of measures described below and an explanatory letter. All participants completed the Locomotion and Assessment scales followed by the Narcissism scale. They then completed a measure designed to assess their positivity. Informed consent was appropriately obtained from the participants. The measures that were used in this study are described in detail below.

#### Regulatory Mode

Participants completed the Italian version of the Regulatory Mode Questionnaire (Kruglanski et al., 2000), composed by two separate 12-item self-report scales designed to measure individual differences in locomotion (e.g., “I enjoy actively doing things, more than just watching and observing”; α = .76) and assessment (e.g., “I often compare myself with other people”; α = .77) on a six-point Likert scale ranging from ‘1’ (Strongly disagree) to ‘6’ (Strongly agree). The psychometric properties, internal consistency, temporal stability, and convergent and discriminant validity, of both locomotion and assessment scales, were previously demonstrated in an extensive research program which included Italian samples (Kruglanski et al., 2000).

#### Narcissism

To measure participants’ narcissism, we used the scale developed by Clarke et al. (2015) based on selected (*N* = 80) items from the Narcissistic Pathological Inventory (Raskin & Terry, 1988) and the Pathological Narcissism Inventory (Pincus et al., 2009). Participants of our study responded to the statements on a six-point Likert scale ranging from ‘1’ (Strongly disagree) to ‘6’ (Strongly agree) aimed at measuring the following 8 dimensions of narcissism: Leadership/Authority (e.g., “I see myself as a good leader”; α = .89), Grandiose Exhibitionism (e.g., “I like to be the center of attention”; α = .88), Manipulative (e.g., “I can read people like a book”; α = .86), Superiority (e.g., “If I ruled the world it would be a much better place”; α = .82), Contingent Self-Esteem (e.g., “It irritates me when people don’t notice how good a person I am”; α = .91), Grandiose Fantasy (e.g., “I often fantasize about being admired and respected”; α = .88), Devaluing the Self (e.g., “I often hide my needs for fear that others will see me as needy and dependent”; α = .87), and Entitlement (e.g., “I can get pretty angry when others disagree with me”; α = .85). The psychometric properties were previously examined by Clarke et al. (2015). The multidimensionality of the narcissism scale was corroborated by explorative and confirmatory factor analyses (Clarke et al., 2015).

#### Positivity

Participants’ positivity was measured through the 8-item (e.g., “I generally feel confident in myself”; α = .85) self-report Positivity Scale (Caprara et al., 2012), with responses on a six-point Likert scale ranging from ‘1’ (Strongly disagree) to ‘6’ (Strongly agree). The psychometric properties of the Positivity Scale were previously examined (see Caprara et al., 2012, for details). The unidimensionality of the Positivity Scale was corroborated by confirmatory factor analysis (Caprara et al., 2012).

### Preliminary Analysis: Validity of Locomotion and Assessment Regulatory Modes, Narcissism Dimensions, and Positivity Measures

To assess the convergent and discriminant validity of regulatory modes, narcissism, and positivity measures, a confirmatory factor analysis (CFA) by means of LISREL 8.5 (Jöreskog & Sörbom, 1996) was conducted with eleven (correlated) latent factors (the locomotion and assessment regulatory modes, the eight narcissistic dimensions, and positivity). The measurement model tested was specified as a partial disaggregation model (Bagozzi & Heatherton, 1994) that use aggregates of items to form two or more indicators per construct. Partial disaggregation models reduce the number of observed variables and the number of parameters being estimated in the models, which accommodates modeling with smaller sample sizes and reduces the likelihood of computational problems. Moreover, the aggregation procedure reduces measurement error in the observed indicators (Bagozzi, 1993; Bentler, 1989). In the present study, for each of the latent construct we computed two manifest indicators using the split-half procedure: each indicator is thus formed from half of the items included in the scales or sub-scales aimed at measuring the constructs considered. Goodness of fit of the model was evaluated via several different indexes: Chi-square, Comparative Fit Index (CFI), and standardized Root Mean Square Residual (RMSR), as recommended by various sources (cf. Bollen, 1989; Tanaka, 1993). The covariance matrix was used as input. CFA results show that the model fit was satisfactory, χ^2^ (154, *N* = 190) = 313.62, *p* = .00; CFI = .97; RMSR = .05. The factor loading values were all significant and above .52, thus demonstrating convergent validity for the constructs. The correlations between latent factors were all statistically less than 1.00 (ranging between .02 and .67), and therefore achieved statistical discriminant validity (Bagozzi, 1994). It is notable that the correlations between latent factors are correlations corrected for attenuation and are expected to be higher than raw coefficients (see Table 1). To further prove discriminant validity of the constructs and control the common method/source biases (due to the cross-sectional design, and, specially, to the all self-report measures used in the present study), we compared the estimated eleven-factor model with two alternative models: one with three latent factors (a unique regulatory mode factor, a unique narcissism factor, and one positivity factor) and one with one latent factor (see Podsakoff et al., 2003, for review on Common Method Biases in Behavioral Research and Recommended Remedies). Results show that the eleven-factor model fits the data better than either the three-factor model (χ^2^ (206, *N* = 190) = 1932.79, *p* = .00; CFI = .69; RMSR = .18), or the one-factor model (χ^2^ (209, *N* = 190) = 2175.09, *p* = .00; CFI = .65; RMSR = .18), thus supporting the distinction between the different constructs used in the present study.

## Mediational Analysis

In order to test the mediation of narcissism in the relationship of regulatory mode with positivity, we estimated a model that includes multiple mediators (the eight dimensions of narcissism) and multiple predictor variables (locomotion and assessment). The analysis was performed using the PROCESS macro for SPSS (Model 4) that can be used to estimate the coefficients in a multiple mediation model with multiple independent variables (Hayes, 2018). We employed Preacher and Hayes’s (2008) procedure to extrapolate estimates of direct and indirect effects. Ninety-five percent CIs were employed and 1000 bootstrapping resamples were run*.*

## Results

Descriptive statistics and correlations between variables are presented in Table 1.

**Table 1:** Summary for means, standard deviations, and correlations between variables

	*M(SD)*	*1*	*2*	*3*	*4*	*5*	*6*	*7*	*8*	*9*	*10*	*11*
1.Leadership/Authority	3.34(.92)	(.89)										
2.Grandiose Exhibitionism	3.07(.91)	.58^***^	(.88)									
3.Manipulative	2.94(.97)	.61^***^	.46^***^	(.86)								
4.Superiority	3.66(.93)	.61^***^	.52^***^	.57^***^	(.82)							
5.Contingent Self-Esteem	3.01(.77)	.13	.27^****^	.10	.10	(.91)						
6.Grandiose Fantasy	3.33(.95)	.36^***^	.31^***^	.30^***^	.39^***^	.61^***^	(.88)					
7.Devaluing the Self	2.68(.85)	.08	.15^*^	.15^*^	.02	.48^***^	.46^***^	(.87)				
8.Entitlement	3.19(.77)	.34^***^	.47^***^	.37^***^	.37^***^	.67^***^	.58^***^	.48^***^	(.85)			
9.Locomotion	4.50(.50)	.37^***^	.27^***^	.16^*^	.24^***^	.02	.17^*^	.02	.12	(.76)		
10.Assessment	3.56(.62)	.26^***^	.30^***^	.27^***^	.20^**^	.54^***^	.49^***^	.33^***^	.56^***^	.16^*^	(.77)	
11.Positivity	4.35(.73)	.35^***^	.32^***^	.17^*^	.35^***^	−.29^***^	−.09	−.33^***^	−.07	.22^**^	−.14^*^	(.85)

Note: ^*^p ≤ .05 ^**^p ≤ .01 ^***^p ≤ .001; In bracket (Cronbach’s Alpha). *N* = 190.

The table shows that positivity was significantly and positively correlated with Leadership/Authority, Grandiose Exhibitionism, Manipulative, Superiority, and significantly and negatively correlated with Contingent Self-Esteem and Devaluing the Self, whereas it was unrelated with Grandiose Fantasy and Entitlement dimensions. Locomotion was positively correlated with Leadership/Authority, Grandiose Exhibitionism, Manipulative, Superiority, Grandiose Fantasy, and unrelated with Contingent Self-Esteem, Devaluing the Self, and Entitlement dimensions. Locomotion was also positively correlated with positivity. Assessment was positively correlated with each dimension of narcissism, and negatively correlated with positivity.

### A Mediational Analysis: Multiple Mediator Model

**Fig. 1:**
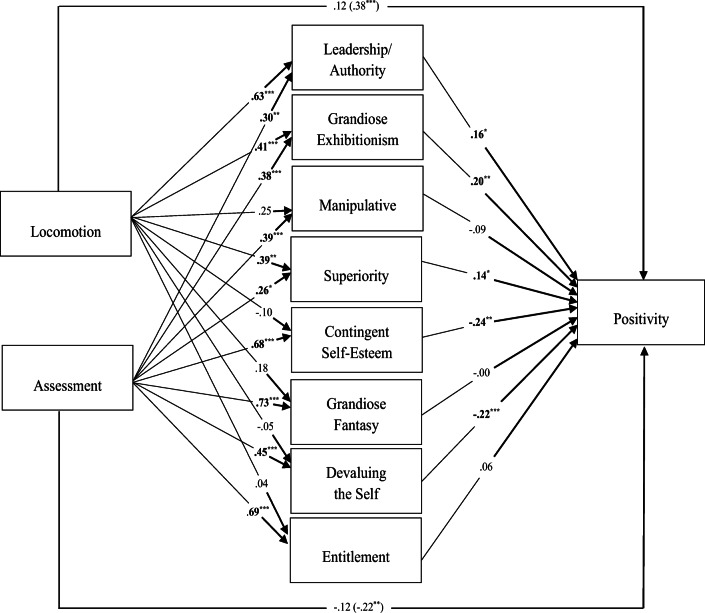
Mediation model of the relationship between locomotion and assessment regulatory mode orientations, narcissism dimensions and positivity. The reported values are unstandardized coefficients. The total effects of mode are included in parenthesis. The estimated total model was significant: *F*(10, 179) = 11.54, *p* < .001, with *R*^2^ = .39. ^*^p ≤ .05 ^**^p ≤ .01 ^***^p ≤ .001

As anticipated, we tested the hypothesized mediating role of narcissism in the relationship between regulatory mode and positivity. Results are summarized in Table 2 and Fig. 1.

**Table 2:** Indirect Effects of Regulatory Mode on positivity through proposed mediators

Mediators	*B*	Boot *SE*	BootLLCI	BootULCI
Mediated effects of locomotion				
Leadership/Authority	.1027	.0511	.0136	.2167
Grandiose Exhibitionism	.0843	.0393	.0268	.1847
Manipulative	−.0220	.0202	−.1088	.0017
Superiority	.0571	.0316	.0107	.1517
Contingent Self-Esteem	.0244	.0247	−.0141	.0906
Grandiose Fantasy	−.0003	.0160	−.0326	.0365
Devaluing the Self	.0109	.0288	−.0402	.0826
Entitlement	.0025	.0118	−.0113	.0472
Mediated effects of assessment				
Leadership/Authority	.0487	.0294	.0047	.1226
Grandiose Exhibitionism	.0780	.0360	.0224	.1665
Manipulative	−.0354	.0279	−.0977	.0127
Superiority	.0371	.0236	.0048	.1114
Contingent Self-Esteem	−.1678	.0617	−.2967	−.0504
Grandiose Fantasy	−.0012	.0568	−.1060	.1141
Devaluing the Self	−.1001	.0408	−.1877	−.0285
Entitlement	.0391	.0691	−.0880	.1872

Note. Coefficients are unstandardized. *N* = 190

As expected and consistent with previous results (Clarke et al., 2015), the dimensions of narcissism most representative of the adaptive form were significantly and positively associated with positivity (Leadership/Authority: *b* = .162, *p* = .025, 95% CI [.021, .304]; Superiority: *b* = .145, *p* = .033, 95% CI [.012, .277]). Consistently with Clarke et al. (2015), Grandiose Exhibitionism also was significantly and positively associated with positivity (*b* = .204, *p* = .002, 95% CI [.077, .331]). The dimensions of narcissism most representative of the maladaptive form were significantly and negatively associated with positivity (Contingent Self-Esteem: *b* = −.245, *p* = .006, 95% CI [−.421, −.070]; Devaluing the Self: *b* = −.221, *p* = .001, 95% CI [−.344, −.098]). As shown in the Fig. 1, as the total effects of both regulatory mode on positivity were significant (Locomotion: *b* = .378, *p* < .001, 95% CI [.174, .581]; Assessment: *b* = −.223, *p* = .008, 95% CI [−.386, −.059]), their direct effects became non-significant after controlling for the mediators (Locomotion: *b* = .118, *p* = .208, 95% CI [−.066, .302]; Assessment: *b* = −.121, *p* = .174, 95% CI [−.296, .054]).

Locomotion was significantly and positively associated with the dimensions of adaptive narcissism, such as Leadership/Authority (*b* = .633, *p* < .001, 95% CI [.391, .876]), Grandiose Exhibitionism (*b* = .413, *p* = .001, 95% CI [.169, .658]) and Superiority (*b* = .395, *p* = .003, 95% CI [.138, .652]). Importantly, there was a significant indirect effect (see Table 2) of locomotion on positivity through Leadership/Authority (Indirect effect = .10, *SE* = .05, 95% CI [.0136, .2167]), Grandiose Exhibitionism (Indirect effect = .08, *SE* = .04, 95% CI [.0268, .1847]), and Superiority (Indirect effect = .06, *SE* = .03, 95% CI [.0107, .1517]), respectively. Therefore, the positive relation between locomotion and positivity appeared to be mediated by adaptive narcissism of leadership, superiority, and grandiose exhibitionism, providing support for H1.

On the other hand, assessment was significantly and positively associated with each of the dimensions of narcissism (Fig. 1; Leadership/Authority: *b* = .300, *p* = .003, 95% CI [.105, .495]; Grandiose Exhibitionism: *b* = .382, *p* < .001, 95% CI [.186, .579]; Manipulative: *b* = .395, *p* < .001, 95% CI [.179, .611]; Superiority: *b* = .257, *p* = .015, 95% CI [.050, .464]; Contingent Self-Esteem: *b* = .684, *p* < .001, 95% CI [.532, .836]; Grandiose Fantasy: *b* = .729, *p* < .001, 95% CI [.537, .922]; Devaluing the Self: *b* = .454, *p* < .001, 95% CI [.267, .641]; Entitlement: *b* = .695, *p* < .001, 95% CI [.546, .844]). Of great interest, there was a significant positive indirect effect of assessment (Table 2) on positivity through Leadership/Authority (Indirect effect = .05, *SE* = .03, 95% CI [.0047, .1226]), Superiority (Indirect effect = .04, *SE* = .02, 95% CI [.0048, .1114]), and Grandiose Exhibitionism (Indirect effect = .08, *SE* = .04, 95% CI [.0224, .1665]). Therefore, the positive relation between assessment and positivity appeared to be mediated by adaptive narcissism of leadership, superiority, and grandiose exhibitionism, providing support for H2. Furthermore, there was a significant negative indirect effect of assessment on positivity through Contingent Self-Esteem (Indirect effect = −.17, *SE* = .06, 95% CI [−.2967, −.0504]) and Devaluing the Self (Indirect effect = −.10, *SE* = .04, 95% CI [−.1877, −.0285]). Therefore, the negative relation between assessment and positivity appeared to be mediated by maladaptive narcissism of contingent self-esteem and devaluing the self, providing support for H3. No significant indirect effects were found through Manipulative, Entitlement and Grandiose Fantasy. The model estimated was overall highly significant, *F*(10, 179) = 11.54, *p* < .001, with *R*^2^ = .39.

## Discussion

Consistent with our predictions, we found that content of narcissism (i.e., adaptive or maladaptive) has mediated the relationship between regulatory mode and positivity. Specifically, both locomotion and assessment orientations were positively associated with positivity through adaptive narcissism. At the same time, assessment was also negatively associated with positivity through maladaptive narcissism: this particular result on assessment is quite consistent with previous results (Boldero et al., 2015) that concluded that the aim of assessment to “do the right thing” can imply different feelings of narcissism in case of success and failure. Therefore, in our case, roles of authority, feelings of superiority and the desire to be the center of attention (i.e., adaptive narcissism), have increased assessors’ positivity; at the same time, the need to be admired by others and the propensity to devalue themselves when they feel unappreciated (i.e., maladaptive narcissism), has reduced their positivity.

Expanding previous research findings (Boldero et al., 2015), the results of the present study confirm the susceptibility of locomotors and assessors to various facets of narcissism, but also outlined how this can affect their ability to see life with a positive outlook. Consistent with recent research on the positive effect of locomotion on well-being (e.g., Di Santo et al., 2020), we see that locomotion promoted positivity through adaptive narcissism. However, assessment also, but not exclusively, promoted positivity through adaptive narcissism. These findings were consistent with previous results on regulatory mode *conjunction positive effects* (Pierro, Giacomantonio, et al., 2012a; Pierro et al., 2018), that is, the beneficial co-presence of the two modes. Successful self-regulation involves both assessment mode, through which the individual thinks deeply about the right course of action, and locomotion mode, through which the individual implements the action; hence, high locomotion, pushed “to go”, makes use of guidance and evaluation control to go “in the right direction” (Pierro et al., 2018; see also Pierro et al., 2006; Pierro, Pica, et al., 2012b). Moving on to the present research, both regulatory modes were found to promote adaptive narcissism and positivity; on the other hand, the simultaneous experience of adaptive and maladaptive narcissism in the assessment mode (Boldero et al., 2015) has been shown to have significant repercussions on positivity.

This work has several limitations that should be noted. We used a sample of university students; thus, we must be particularly cautious in generalizing the results to other populations. Furthermore, our data was collected from the same source and using the same method: all data derived from self-report measures and cross-sectional design. Thus, on the one hand, data and findings may be subject to common method/source biases, that may inflate relationships between variables, and, on the other hand, do not even allow to delineate the causality of the relationships found. Although we controlled the common method/source bias comparing alternative models via CFA, and confirming the discriminant validity of the measures used, further research should also profitably use multitrait-multimethod matrix (MTMM; Fiske & Campbell, 1992) to better address this issue. Furthermore, future research should provide confirmation for our hypothesis using longitudinal or experimental designs with manipulation of variables (e.g., Avnet & Higgins, 2003). However, we can be cautiously confident in our results since previous studies found consistent relationships between regulatory mode and narcissism (Boldero et al., 2015), and positive affects (Kruglanski et al., 2000), as well as between narcissism and self-worth (Clarke et al., 2015); we thus determined the proper order of the variables in our proposed mediation model upon the relationships previously found.

Beyond these limitations, these results can have theoretical implication linked to our attempt to connect previous conclusions (Boldero et al., 2015; Clarke et al., 2015) and examine a model that sees self-regulation, narcissism, and positive thinking as potentially linked processes. Certainly, future research can examine these relationships further. Or, given that narcissism was only a partial mediator, future studies should continue exploring other potential mediators. Practical implications are related to the possibility of stimulating (e.g., Avnet & Higgins, 2003) self-regulatory factors that increase individuals’ susceptibility to different forms of narcissism. Of course, we must duly emphasize that narcissism is still at the center of a large debate in the clinical and social literature (see, for example, Di Pierro & Madeddu, 2018). The debate stems from the high complexity of the narcissistic syndrome, and there is still widespread disagreement on its definition and measurement, as well as the identification of its central characteristics, so we find an abundance of conflicting results and theoretical perspectives in the literature, as was also argued by Morf and Rhodewalt (2001). However, previous research (e.g., Cai & Luo, 2018; Clarke et al., 2015) has shown that narcissism can be “adaptive” if it brings positive psychological benefits to the individual. Therefore, by considering locomotion and assessment as possible self-regulatory bases of narcissistic forms, we may have in mind that stimulating both can lead to adaptive forms of narcissism. However, assessment has tendencies towards threatening and continuous evaluation with various behavioral consequences (e.g., Livi et al., 2014), which can also lead to experience maladaptive outcomes, appearing the self-regulatory factor that stimulates different presentations of narcissism in individuals (see also the argument of Boldero et al., 2015).

It cannot be excluded that other factors may intervene to favor maladaptive outcomes, linked to the personal history of the individual; for example, child maltreatment has been shown to increase the risk of negative psychological outcomes and maladaptive responses (Pompili et al., 2014). Thus, in the study of the development of narcissistic self-regulation and its effects, personal factors of the individual should be considered, for example relating to childhood experiences (e.g., Otway & Vignoles, 2006).

Turning to the current research, a distinction was confirmed in the positive effects of adaptive versus maladaptive narcissism. Therefore, this study offers some insight into how having (or possibly stimulating) locomotion mode, assessment mode, or both, and understanding the forms of narcissism linked with them, can help individuals live their lives with greater positivity.

## Conclusion

Results of this study show that regulatory modes were associated with increased or decreased positivity through narcissism. Specifically, locomotion was associated with greater positivity through adaptive narcissism. On the other hand, assessment was associated with increased and decreased positivity through adaptive and maladaptive narcissism, respectively. These findings raise important questions about the role of self-regulation in predicting types of narcissism and related well-being. In conclusion, what emerges from our findings is that regulatory modes and adaptive narcissism can help people approach their life positively.

## Data Availability

The datasets generated during and analysed during the current study are available from the corresponding author on reasonable request.
